# Bridging consciousness and AI: ChatGPT-assisted phenomenological analysis

**DOI:** 10.3389/fpsyg.2025.1520186

**Published:** 2025-05-29

**Authors:** David Martínez-Pernía, Alejandro Troncoso, Sergio E. Chaigneau, Nicolás Marchant, Antonia Zepeda, Kevin A. Blanco-Madariaga

**Affiliations:** Center for Social and Cognitive Neuroscience (CSCN), School of Psychology, Universidad Adolfo Ibáñez, Santiago, Chile

**Keywords:** mixed-methods studies, ChatGPT-assisted phenomenological analysis, phenomenology, neurophenomenology, artificial intelligence, ChatGPT, experimental phenomenology

## Abstract

**Background:**

Mixed-method studies require adaptation to the era of big data in quantitative research, seeking scalable approaches that can analyze extensive qualitative datasets while preserving the depth and nuance inherent in the study of consciousness on a broader scale.

**Objective:**

This study aimed to leverage ChatGPT, renowned for its descriptive generation proficiency, to perform a phenomenological analysis.

**Methodology:**

Our research followed four key stages: (1) Preparation of Phenomenological Data, where transcriptions were refined to align with the research question; (2) Individual Analysis, where ChatGPT highlighted experiential nuances from each participant; (3) Global Analysis, synthesizing insights from individual narratives temporally and transversally; and (4) Structure of the Experience, which synthesized the elemental components of shared experiences. Custom prompts, tailored for each stage, ensured alignment and precision in capturing the experience dimensions.

**Results:**

ChatGPT showcased a sophisticated processing capability of human experiences, effectively organizing themes that reflect the intensity of sensations and variations in empathetic encounters. The tool’s proficiency in thematic organization provided a phenomenologically-grounded processing of data, highlighting how individuals engage with and are affected by stimuli.

**Discussion:**

Our findings highlight ChatGPT’s potential in consciousness studies, transforming raw input into detailed phenomenological accounts. ChatGPT combines precision with scalability, making it a compelling tool for researchers exploring the intricacies of human experiences. Further research is essential to better understand AI’s capacity in phenomenological analysis and to strengthen the methodological framework, ensuring it effectively captures the nuances and depth of phenomenological inquiry.

## Introduction

The exploration of consciousness and the cosmos represents two compelling frontiers of human inquiry, each inviting our curiosity and investigation, yet holding distinct positions in scientific study (Chalmers, 1995; Metzinger, 2004). While the cosmos has been traditionally studied through empirical methods focused on external, quantifiable measurements, the study of consciousness has necessitated a more complex approach (Varela, 1996). This methodological contrast arises from the nature of each phenomenon: the cosmos, as an physical and objective reality, allows for examination through a reductionist lens, relying solely on observable phenomena. Consciousness, on the other hand, signifies an inherently subjective and personal phenomenon, where the reductionist approach alone proves inadequate (Levine, 1983; Nagel, 1974; Searle, 1997; Varela, 1996; Thompson, 2007).

This fundamental difference has led to the recognition that consciousness must be studied with methodological frameworks capable of accounting for its dual character. On one hand, consciousness is a biological and neurophysiological process that can be measured empirically. On the other, it is also an irreducibly qualitative phenomenon—the “what it is like” dimension of experience—that requires tools sensitive to subjectivity. As such, recent decades have witnessed growing interest in mixed-methods approaches that combine the empirical strength of neuroscience with the descriptive richness of phenomenology (Varela and Shear, 1999; Petitmengin and Bitbol, 2009). This paradigm shift has inspired a rethinking of methodology in the cognitive sciences, calling for a balanced engagement with both third-person data and first-person accounts (Creswell and Clark, 2017; Varela and Shear, 1999; Johnson and Onwuegbuzie, 2004; Teddlie and Tashakkori, 2009).

Traditional methods for accessing subjectivity in the sciences of consciousness have relied heavily on self-report questionnaires (Gallagher and Sørensen, 2006). These instruments—such as Likert scales or discrete categories like “fear,” “joy,” or “sadness”—allow for large-scale data collection and statistical generalization. However, they are often criticized for simplifying complex subjective phenomena into standardized, quantifiable data points, thereby aligning with a methodological behaviorism that prioritizes operationalization over lived experience (Creswell and Clark, 2017; Olivares et al., 2015). The depth of human consciousness, its embodied nature, and its context-sensitive dimensions often escape the reach of such methods.

In response to these limitations, scholars have called for a deeper exploration of first-person perspectives, shifting away from introspection in the psychological field and anchoring it in the phenomenological tradition (Zahavi, 2013). Phenomenology, initiated by Husserl and developed by thinkers such as Merleau-Ponty, and more recently by Gallagher and Zahavi, focuses on describing consciousness from within —on articulating the structures of lived experience in their intentional, temporal, and embodied dimensions (e.g., Merleau-Ponty, 1945; Brentano, 2012; Husserl, 2001; Reinach, 1968; Gallagher and Zahavi, 2020; Zahavi, 2021). This approach treats consciousness not merely as an object of study but as the very medium through which the world appears and becomes meaningful.

One significant attempt to integrate phenomenology and neuroscience is Francisco Varela’s neurophenomenological program (Varela, 1996). His proposal sought to bridge third-person measures of brain activity with first-person descriptions of experience, based on a reciprocal constraint model. Since then, several lines of research have emerged to expand this method, including neurophysiophenomenology (Colombetti, 2013), cardiophenomenology (Depraz and Desmidt, 2019), and neuro-physio-socio-phenomenology (Froese, 2015). More recently, methodologies using mobile brain and body imaging (MoBI) have attempted to preserve ecological validity while integrating phenomenological interviews in naturalistic contexts (Troncoso et al., 2023).

Despite the promising potential of mixed-methods approaches, integrating quantitative and qualitative methodologies presents notable challenges—particularly in data analysis. Reconciling these approaches requires careful consideration of sample size. In recent years, a growing consensus in the scientific community has underscored the need for larger sample sizes to improve scientific validity and support replicability (Button et al., 2013; Marek et al., 2022; Simmons et al., 2011). This shift is echoed in computational statistics, where robust and generalizable models depend on diverse, high-volume datasets (Goodfellow et al., 2016; Varoquaux, 2018). In such frameworks, hundreds or even thousands of participants may be required to detect subtle effects and accommodate the complexity of human phenomena. While initially driven by statistical aims, this emphasis on scale also reflects a commitment to capturing the richness and variability of the subject matter—an objective that aligns with the aims of qualitative methodology.

However, incorporating large sample sizes into qualitative studies poses a substantial methodological challenge. Although such scaling enhances reliability in quantitative terms, it conflicts with the intensive, meaning-oriented nature of traditional qualitative practices. Methods such as phenomenological meaning analisys and thematic analysis are labor-intensive and time-consuming (Guest et al., 2006; Morse, 2015; Sandelowski, 2000). As sample sizes grow, so do the risks of losing the nuance and contextual sensitivity central to phenomenological inquiry. The iterative process of coding, theme development, and narrative interpretation can become strained, potentially diluting reflexivity and researcher engagement (Maxwell, 2010; Morse and Niehaus, 2016). Moreover, the pursuit of thematic saturation becomes more difficult as the volume of data increases, challenging the feasibility of preserving the subtlety and individuality of lived experience within large-scale analyses (Guest et al., 2006; Maxwell, 2010).

In this context, there is an urgent need for methodological innovation. The goal is not to abandon phenomenological rigor but to develop tools that allow for the preservation of its insights at scale. As the scientific landscape demands larger samples, and reproducible findings, it is imperative to explore ways to make qualitative research more scalable without falling into reductionism. One possible solution lies in the use of artificial intelligence (AI), particularly natural language processing (NLP), to assist in the interpretation of phenomenological data.

To address the challenge of scaling phenomenological analysis without sacrificing its descriptive depth, this paper explores the potential of large language models (LLMs)—specifically ChatGPT (GPT-4, OpenAI)—as a tool for analyzing first-person qualitative data. These models are trained on vast linguistic corpora and are capable of generating coherent, context-sensitive language outputs based on complex prompts (Brown et al., 2020; Vaswani et al., 2017; Bengio et al., 2021). For this study, we used ChatGPT Plus, the paid web-based version that provides user access to the GPT-4 architecture. ChatGPT, as implemented here, operates as an autoregressive language model that processes language sequentially, using prior context to generate semantically meaningful outputs. Originally designed for next-word prediction, GPT models have since been adapted to perform a range of language tasks, including text generation, translation, summarization, and textual analysis. Through unsupervised training on large and diverse datasets, these models acquire the capacity to interpret linguistic structures across multiple levels of meaning. Enabling the automation of qualitative analysis while preserving phenomenological granularity. Building on established NLP techniques such as sentiment analysis (Pang and Lee, 2008), topic modeling (Blei et al., 2003), and semantic analysis (Turney and Pantel, 2010), our methodology seeks to extract recurring patterns, affective tones, and conceptual distinctions from rich, narrative data. This approach builds upon the growing literature in computational linguistics and AI (Bird et al., 2009; Jurafsky and Martin, 2008), while also staying grounded in the philosophical foundations of phenomenology. Rather than treating LLMs as mere tools of classification or categorization, we conceptualize them as potential mediators of intersubjective understanding—systems that can assist researchers in navigating the complexity of human experience without replacing human judgment. Inspired by recent methodological advances in descriptive phenomenology (Berkovich-Ohana et al., 2020; Nave et al., 2021; Martínez-Pernía et al., 2023; Martínez-Pernía, 2022; Troncoso et al., 2024; Troncoso et al., 2025; Vergara et al., 2022; Zepeda et al., 2025), our approach maintains fidelity to the lived, intentional structures of experience, even as it embraces computational assistance.

This manuscript offers a proof of concept for a new methodological path—one that neither abandons the phenomenological tradition nor ignores the possibilities of AI-enhanced description. It represents a first step in that direction. As the sciences of consciousness evolve, the growing demand for larger sample sizes challenges qualitative research to adopt more scalable methodologies. Without such innovation, mixed-methods risk losing relevance in an increasingly data-driven landscape. This study responds to that challenge by integrating the depth of qualitative inquiry with the scalability of AI tools like ChatGPT, aiming to advance interdisciplinary research through scalable mixed-methods frameworks in the science of consciousness.

## Methodological overview

### Experimental procedure and phenomenological data collection

This study analyzes a previously acquired dataset, originally collected and analyzed using human-driven phenomenological methods (Martínez-Pernía et al., 2023). We provide a brief summary of the experimental setup here, and refer readers to that publication for full methodological details.

#### Participants

The study involved 28 adults who met specific criteria (mean age = 29.6 ± 6.6 years). Inclusion criteria required participants to have no clinical history of cognitive, neurological, or psychiatric disorders and to possess normal or corrected-to-normal visual acuity. Participants provided written informed consent. The study adhered to the principles of the Declaration of Helsinki and was approved by the Scientific Ethics Committee.

#### Construction and validation of the emotional stimuli

The emotional stimuli, specifically empathy for pain stimuli, were created and validated using established methods in affective neuroscience. A total of 12 scenes depicting intense physical accidents during extreme sports were used for validation. These scenes were selected from online sources under Creative Commons licensing. Each scene featured a sportsperson skillfully practicing a sport, losing balance, and experiencing a heavy impact, but they did not depict dismemberment, disfigurement, or death. These scenes were validated by 65 university students using the Self-Assessment Manikin scale, which assessed valence, arousal, and dominance of emotional reactions. A compilation of seven scenes was assembled to create the ultimate video, which had a duration of 60 s and was viewed by all participants.

#### Procedure

**Figure 1:**
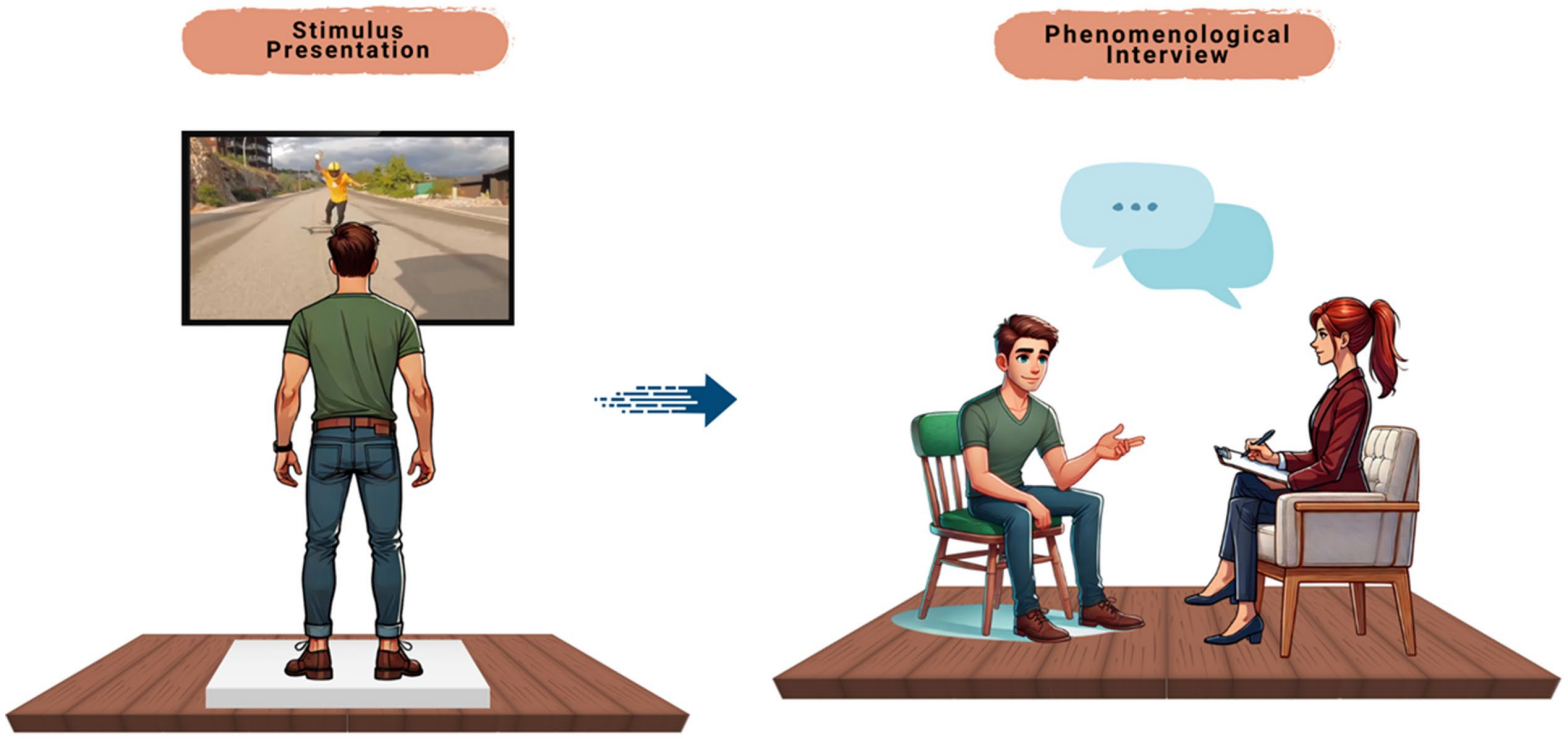
The figure illustrates the two main stages of data collection. On the left, the participant views an emotionally salient video stimulus while standing, eliciting a first-person embodied response. On the right, the same participant engages in a phenomenological interview guided by a trained phenomenological researcher, aimed at eliciting detailed verbal descriptions of their lived experience during the stimulus presentation. This process constitutes the core of the first-person data collection for subsequent AI-assisted analysis.

Participants were asked to stand motionless 1 meter from a 40-inch screen displaying the video. After viewing the video, each participant underwent a phenomenological interview conducted by a researcher (Figure 1).

#### The phenomenological interview

The goal of the phenomenological interview was to examine the empathic experience with a particular focus on the embodied, multi-layered dimensions (bodily sensations, emotions, motivations, and cognitions) and temporal aspects of the empathic experience from the participant’s perspective while watching the physical accidents of the athletes. The interviews were partially guided by the principles of micro-phenomenological interviews and evocation to elicit pre-reflective descriptions and vivid descriptions (Please see Supplementary material for detailed description of the interview).

### The phenomenological analysis assisted by ChatGPT

In order to direct the phenomenological analysis with the aid of ChatGPT, we integrated the primary steps outlined in Giorgi’s descriptive phenomenological psychological method (Englander and Morley, 2023; Giorgi et al., 2017) and Petitmengin’s micro-phenomenological approach (Petitmengin et al., 2019; Valenzuela-Moguillansky and Vásquez-Rosati, 2019). The guiding criterion for crafting this ChatGPT-assisted analysis was to maintain a descriptive phenomenological orientation, adapting its core principles to a format compatible with AI-supported interpretation. At this point, it is important to note that the phases of epoché and phenomenological reduction were not implemented, as these are not cognitive operations that can be meaningfully performed by AI systems. Nevertheless, this omission does not undermine the phenomenological validity of the analysis. Although in the discourse surrounding applied phenomenology, the necessity of employing the epoché and reduction is often endorsed, a closer examination reveals that this understanding may be flawed. The crux of the matter lies in understanding that the applied phenomenology is not strictly tied to the epoché and reduction (Zahavi, 2021). It is not these techniques themselves that define phenomenology but rather the pursuit of grasping the ‘how’ of experience. A more direct method of descriptive analysis can suffice to uncover the rich textures of experience. This descriptive approach is consistent with the original orientation of phenomenology toward experience as it is encountered (Reinach, 1968; Husserl, 2001; Brentano, 2012; for more information see Zahavi, 2021). In this sense, LLMs such as ChatGPT are particularly well-suited for assisting in descriptive phenomenological analysis, as they excel in recognizing narrative structure, extracting thematic patterns, and preserving semantic nuance.

Given this potential, it is important to consider certain methodological aspects associated with the configuration of the GPT-4 architecture accessed via the paid web-based interface (ChatGPT Plus), particularly those that influence the phenomenological quality of the output. One such aspect is the internal *temperature* parameter, which modulates the probability distribution of token sampling and thereby determines the degree of stochastic variability in the generated text (Brown et al., 2020; Holtzman et al., 2020). Although OpenAI has not officially disclosed the default value for ChatGPT-4, it is widely presumed to be approximately 0.7. By using this default setting in the user-facing version of ChatGPT-4—rather than adjusting it via API—we maintained a balance between textual coherence and creative variation. This controlled spontaneity proved useful for generating nuanced descriptions essential to phenomenological inquiry, while also ensuring consistency in thematic identification and structural analysis.

**Figure 2:**
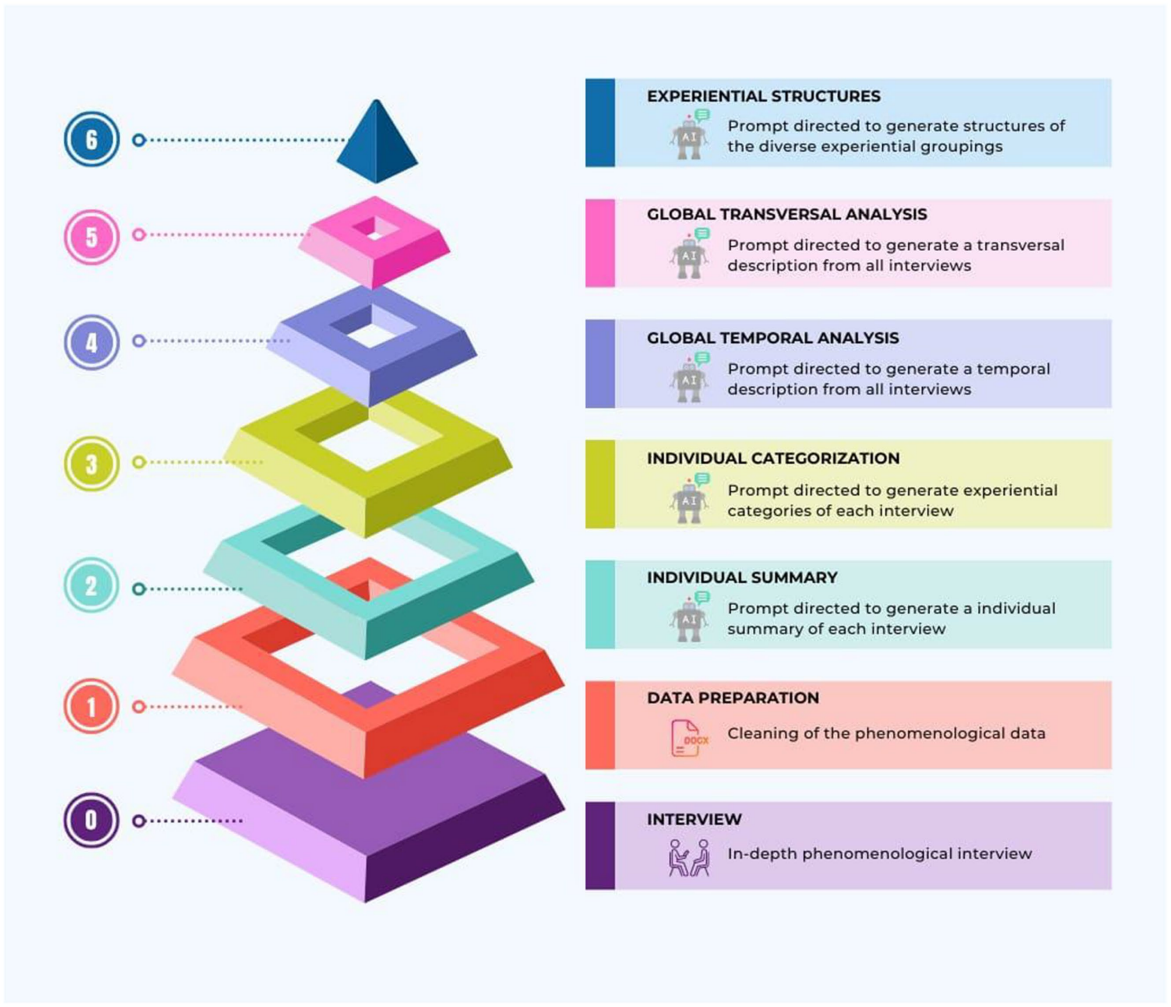
Structured progression in ChatGPT-assisted phenomenological analysis. The diagram’s pyramidal structure illustrates the process of AI-assisted phenomenological inquiry, clearly delineating the transition from qualitative to computationally enhanced phases. Phase 0 anchors the research in a qualitative foundation, where phenomenological interviews gather comprehensive experiential data. From Phase 1 onwards, the ChatGPT-assisted qualitative analysis commences, methodically filtering the data for in-depth interpretation. This represents a shift from purely qualitative interviews at the base to an integrated AI-supported analytical approach, utilizing ChatGPT to identify, organize, and synthesize the experiential dimensions of the collected narratives.

Next, we will outline the traditional phenomenological analysis performed by humans and describe how this approach is adapted for AI analysis. Our methodology is divided into four primary stages. Stage 1, focusses on phenomenological data cleaning (see Step 1 in Figure 2). Stage 2 comprises individual analysis for each participant’s interview (see Steps 2 and 3 in Figure 2). Stage 3 addresses the global analysis of all interviews combined (see Steps 4 and 5 in Figure 2). Finally, Stage 4 summarizes the phenomenological structure of the experience across all analyzed interviews (see Step 6 in Figure 2).

#### Step 1: preparation of the phenomenological data

After the completion of the 28 interviews, they were transcribed into the DOCX format. To provide clear direction for ChatGPT’s analysis, portions of the interviews not relevant to the research objective, such as experiences unrelated to video exposure time or social interactions between the interviewer and participant, were excluded. This methodological step aims to ensure that the data analysis is strictly related to the research question. In addition, all personal data of each participant were properly anonymized.

#### Step 2: individual summary

Building on the prepared data from Step 1, our next step involved extracting a concise overview to capture the essence of the entire described situation of each participant. With ChatGPT, our primary aim was to summarize the most critical aspects of participants’ experiences, especially those linked to pre-reflective dimensions, defined as the implicit, direct experience that exists before self-consciousness or analytical interpretation. These dimensions, following the research question included bodily sensations, emotions, motivations, and cognitions, all mapped according to their temporal progression.

To generate an individual summary for each participant, we entered each interview into ChatGPT’s chat interface independently, using a standardized prompt to ensure internal consistency across cases.

*“Describe in detail and in an integrated manner the most relevant aspects of what the interviewee said, considering the following: bodily sensations, emotional states, intentions, attention, and cognitions. Order the description according to the temporal phases related to the fall that occurred in the video (for example, before, during, and after the fall). The word “I:” represents the interviewer, and the word “E:” represents the interviewee.”*

Given the constraints imposed by the input word limit of ChatGPT-4 and the potential evolution in the quality of data across interviews, a strategic approach was adopted. We divided the interviews into three distinct groups, with each group containing the summaries corresponding to each participant, ensuring that the maximum word limit allowed by ChatGPT was not exceeded. The grouping of the interviews was randomized to prevent early interviews from influencing the analysis of the group containing the latest interviews, considering the subtle changes that can occur when conducting a large-scale interview collection.

#### Step 3: individual experiential categories

Following the third methodological step, the methodological objective of this phase for the ChatGPT analysis was to identify phenomenologically relevant experiential categories and subcategories based on the participants’ accounts. These categories and subcategories served as tools for comprehending diverse phenomenological dimensions and their dynamics throughout the video. In addition, we also ensured that the analysis remained closely intertwined with the provided interviews to prevent potential errors or misinterpretations generated by the model (hallucinations). To maintain this connection, we requested the inclusion of exemplary quotations accompanied by their corresponding paragraph numbers.

In order to organize all the data and enable the researcher to easily access the different components requested in the prompt, as well as their interpretation, we ask ChatGPT to arrange it in a 7-column table for each individual interview. The following prompt was used:

*“Create a 7-column table to provide a detailed description (bodily sensations, emotional states, intentions, attention, and cognitions) of the following interview. Analyze each paragraph of the interview. In the table, write a category of experience in the second column and a subcategory of experience in the third column. In the first column, indicate which moment the paragraph refers to relative to the time of the subject’s fall (*e.g.*, before the fall) In the fourth column, add a short phrase that describes the content. In the fifth column, include an example quote from the interview. In the sixth column, add a definition of the subcategory. In the seventh column, note the code associated with the paragraph (indicated by “<>“). The word “I:” represents the interviewer, and the word “E:” represents the interviewee. Use your own way to group the categories.”*

#### Step 4: global temporal analysis

**Figure 3:**
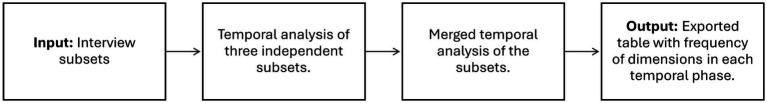
Utilizing three subsets of the summary of 28 interviews, a temporal independent analysis per subset was performed. After, the three analyses were merged into a global analysis. Output generated a table displaying the frequency of samples with specific experiences in each temporal phase.

In Step 4, we used ChatGPT to conduct a global analysis of the temporal structure of the experience—referred to in micro-phenomenological terms as “diachronic analysis” (Petitmengin et al., 2019). This process focused on identifying, categorizing, and relating experiential elements according to their chronological unfolding. To develop this global temporal analysis using ChatGPT, we followed a sequential procedure divided into three sub-steps (Figure 3), using standardized prompts to ensure internal consistency across interview subsets.

First, we introduced ChatGPT’s summaries of the 28 interviews from Step 1 into the chat interface. To overcome ChatGPT’s word limitation, we divided the summaries into three sets, each comprising 10, 10, and 8 interviews, respectively. We then requested ChatGPT’s input for each group in a CSV table format. This tabular format not only facilitated data organization but also served as the foundation for subsequent analyses. The following prompt was used:

*“Create a summary of the temporal phases of the following interviews in a table. Then, provide the summary in CSV format.”*

Second, since a comprehensive temporal analysis required the entire sample, we continued collecting all the grouped table summaries in a single iterative process with ChatGPT. For this purpose, we employed the following prompt:

*“Perform a global analysis of the temporality of the following interviews.”*

Third, to define the primary experiential dimensions used by ChatGPT to describe each temporal phase in terms of frequency, we requested a synopsis of these dimensions during each phase. Additionally, we sought the participant numbers associated with these aspects. This request was formulated using the following prompt.

*“Summarize the following in a table that can be exported to CSV format, indicating the total number of subjects experiencing each aspect.”*

#### Step 5: global transversal analysis

**Figure 4:**
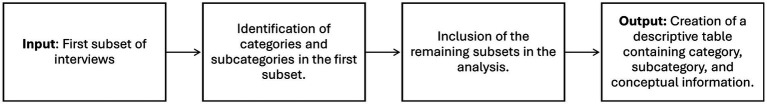
Illustrates global transversal analysis, beginning with the input of a subset of the summary of 10 interviews. Within the initial subset, categories and subcategories are identified. All remaining subsets are integrated into the initial analysis. As a final output, a descriptive table is generated, presenting category, subcategory, and its conceptual definition.

While the prior step employed a phenomenological analysis centered on the diachronic aspects of experience, this phase adopts a parallel approach (Petitmengin et al., 2019) with a distinct focus. Here, the emphasis is on identifying and interacting with experiential categories that could cut across the entirety of the experience, independent of their chronological context. Bearing this in mind, the phenomenological analysis assisted by ChatGPT used the summary of the 28 interviews. We employed a similar procedure for temporal analysis by dividing the interviews into three sets, using standardized prompts to ensure internal consistency across interview subsets (Figure 4).

Firstly, ChatGPT was asked to identify experiential categories and subcategories and its conceptual definition in the first group of interviews.

*“Analyze and group into experiential categories. To do so, describe experiential categories, subcategories.”*

We then carried out a thorough analysis, incorporating categories from each subsequent group into the initial iteration established with the first group. The following prompt was used in this analysis:

*“Using the same structure, add the following participants and, if necessary, include another category.”*

#### Step 6: structure of the experience

In the final phase of the phenomenological analysis, the primary goal is to capture the essential nature of the lived experience by identifying its structural components (Englander and Morley, 2023; Giorgi et al., 2017; Petitmengin et al., 2019; Valenzuela-Moguillansky and Vásquez-Rosati, 2019). This resulted in a holistic and integrated understanding that may encapsulate the very essence of the phenomenon. This phenomenological approach accomplishes this through a meticulous process of abstraction, wherein non-essential elements are stripped away, leaving behind a pure and representative structure of the phenomenon under study.

**Figure 5:**
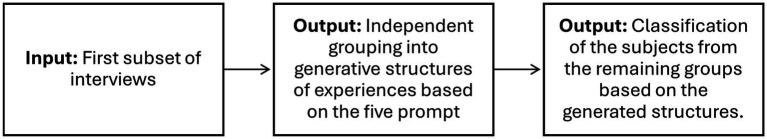
Initially, ChatGPT was fed with the first group of interviews to categorize with generative structures of experiences based on the five different prompts that range from lower to higher control. Subsequently, the determination of the classification of the remaining interviews was conducted.

To guide ChatGPT’s analysis, we employed two distinct modes of instruction: Broad Prompting and Targeted Prompting, with the intention of transitioning from more open-ended structures to ones that are tightly defined across five distinct prompts (Figure 5). Broad Prompting allows ChatGPT significant autonomy, presenting it with open-ended or generalized requests. This approach is designed to facilitate the emergence of structures in a free-form manner, without the constraints of predefined categories or specific presuppositions. In contrast, Targeted Prompting directs ChatGPT’s outputs by providing it with detailed and directive criteria. This prompt emphasizes the use of fixed parameters, ensuring that the outputs conform to specific, predetermined structures. For this purpose, we employed the following prompts:

##### Open-ended prompt

To initiate the analysis, we requested ChatGPT to categorize subjects using an open-ended approach. Specifically, we inputted: *"Group the participants according to their type of experience of the subject’s fall. Include the number of individuals per group. Maximum of 3 grouping groups.”*

##### Creative classification prompt

For the second structure, we directed ChatGPT toward a more inventive method of group formation. Our instruction was: *“Group the participants* into a maximum of 3 groups *using a creative classification.”*

##### Empathy experiences prompt

For the third structure, we asked it to describe the empathic experience based on its own definition of empathy, using the following prompt “Group them into a maximum of 3 groups based on their empathic experience.”

##### Empathy types prompt

Fourthly, we asked ChatGPT to classify subjects into types of empathy according to its internal knowledge base, using the following prompt: “Group the participants into a maximum of 3 categories based on their type of empathy.”

##### Pre-defined empathy prompt

Concluding the series, we directed ChatGPT to use a specific definition from our prior research article (Martínez-Pernía et al., 2023) for categorization. We prompted: *“Group the subjects according to their type of empathy: Self-Centered Empathy: This group refers to individuals who focus on their own bodily sensations and have a motivation toward self-protection or avoidance. Other-Centered Empathy: This group includes individuals who express concern and their affection is focused on the other person. They have a motivation toward helping or caring.”*

For each categorization, after the main prompt, ChatGPT was instructed to include the remaining subjects from the other interview groups using the subsequent prompt.

*“Add the following subjects to the classification.”*

#### Prompts selection criteria

It is important to note that, while ChatGPT can be a useful tool for text analysis and pattern identification, the supervision and guidance strategy of a human researcher was used. ChatGPT’s capabilities can be used to accelerate and automate parts of the analysis process, but the researcher still needs to verify and validate the results, and make their own understanding from the data.

Six primary criteria were employed for selecting prompts. Firstly, a requirement was established for prompt consistency across interviews (Steps 2 and 3) and groups of interviews (Steps 4 and 5). This involved ensuring that the results obtained maintained uniformity in terms of their descriptive structure. By adhering to this criterion, the aim was to achieve coherence and comparability in the generated responses across multiple interview instances. Achieving this consistency required a process of prompt optimization. During the initial stages, we tested various prompts to ensure they produced consistent and coherent outputs across all interviews. For example, earlier prompts sometimes resulted in outputs where tables and summaries were formatted differently for each participant, compromising the comparability of the results. Once a stable prompt was established, it was applied uniformly throughout the analysis. Secondly, the prompt selections were made considering that the ideal responses would be obtained with minimal iterations with ChatGPT. Thirdly, the prompts were meticulously selected to elicit a subjective sense of coherence from the investigators, aligning with their comprehension of the initial interviews. This approach aimed to encourage prompts that resonated with the investigators’ personal understanding. Fourthly, we ensured that the analysis remained closely intertwined with the provided interviews. To maintain this connection, we requested the inclusion of exemplary quotations, along with their corresponding paragraph numbers. Additionally, as a fifth criterion to regulate the degree of experiential structure in Step 6, we instructed ChatGPT to provide a maximum of three experiential structures. This measure was taken to minimize the potential for over-classification while maintaining control over the quantity of experiential information. Moreover, as a sixth consideration, the balance between prompt control and flexibility was adjusted based on the specific step. For instance, in steps 2 and 3, maintaining a high level of control was crucial to ensure consistency across interviews. However, in the global analysis, providing more flexible prompts to ChatGPT without excessive control instructions proved to be effective.

## Results

### Individual summary

The results of this analysis provide a concise summary of the interviews by identifying the dimensions of the experience (e.g., bodily feelings) and describing the temporality of the experience. Each participant’s description of the temporal aspects of their experience aligns with the specific experiential dimension that was explored.

The paragraphs below present an example of the summary of ChatGPT’s response in the given topic, subject 24 (to access the complete interview of Subject 24, please see the Supplementary material).

*“Before the fall:*

*The interviewee expresses bodily sensations of tension, especially in their ankle due to previous experiences of intense sprains. This feeling of tension intensifies when they see someone fall and twist their joint, causing them pain and even nausea. Regarding the videos, the interviewee describes a sense of anxiety and physical discomfort when watching falls that are more severe and violent than a simple sprain. They worry about the potential serious injuries that the individuals in the videos could suffer, such as exposed fractures or spinal damage. The interviewee shows an intention to protect themselves and avoid watching the videos due to the emotional intensity they generate.*

*During the fall:*

*The interviewee describes a negative and maximum intensity sensation in the video of the wall climbing. They also mention other videos of falls in skateboarding and skiing, but consider the climbing video to be the most intense. During the viewing of the videos, the interviewee experiences sensations of muscular tension in different parts of their body. The muscles in their face, jaw, shoulders, and hands are tense. Their face shows signs of tension as they clench their teeth and jaw. Additionally, they feel sweat in their hands and keep them clenched as an instinctive protective reaction.*

*After the fall:*

*After the fall, the interviewee describes a decrease in muscular tension but still experiences a generalized sense of tension in their body. They mention that the muscular tension is lower in the skateboarding videos compared to the climbing video. There is also a sense of adrenaline and danger observed in the video of the girl walking on a tightrope, due to the height and the fact that she is wearing heels. The interviewee feels an anxious anticipation and experiences a sense of relief when the girl manages to grip and avoid the fall. This feeling of relief corresponds to a rapid inhalation and a sensation of “aaahhh.””*

### Individual experiential categories

The result showcases ChatGPT’s proficiency in comprehending a range of experiential dimensions by presenting well-founded definitions, illustrative quotes, and the corresponding paragraphs where ChatGPT successfully identified them. The following table is an example of the same subject above (Table 1).

**Table 1:** Individual summary.

Time	Category	Subcategory	Content	Example quote	Definition	Code
Before	Emotional States	Fear and apprehension	Feeling fear and apprehension when witnessing falls, particularly anticipating negative outcomes.	“I panic...I do not know, I do not feel that I can support my hands...that you can see an exposed fracture, that aaaah!”	Fear and apprehension experienced when anticipating negative outcomes and severe injuries resulting from falls.	<J242>
		Disgust	Expressing disgust and aversion toward watching falls, particularly feeling nauseous and repelled by injuries.	“I panic falling...the pain is so intense...I’ve had so many sprains in my ankle, and the pain is so intense...It even makes me nauseous.”	Disgust and aversion toward watching falls, triggered by the anticipation of severe injuries and feeling physically repelled by the sight of injuries.	<J242>
During	Bodily sensations	Sweating	Noticing increased sweating, particularly in the hands, as a physical reaction to feeling discomfort and distress.	“The sweating of hands was not even an anguish in front because it was different...they were all wet...my hands, I do not remember if I held them...because they were sweaty.”	Increased sweating, particularly in the hands, as a physical reaction to discomfort and distress experienced while watching falls.	<J2445>
		Muscle tension	Experiencing muscle tension, particularly in the face, neck, shoulders, and hands, as a reaction to distress.	“I clenched them a lot: the teeth, the jaw...it was marked even the teeth in my mouth...neck, tense neck, face, the muscles all clenched;... I felt tense all the time...the hands, I do not remember if I held them.”	Muscle tension experienced throughout the body, particularly in the face, neck, shoulders, and hands, as a reaction to distress and discomfort triggered by watching falls.	<J2461>
During	Attention	Hyperfocus	Demonstrating hyperfocus on specific details of falls, particularly anticipating injuries and negative outcomes.	“The first thing that comes to my head, was ok: the babe is going to fall, the rope is not going to work...I did not even want to state or that I was going to have her dangling...”	Hyperfocus on imagining potential injuries and distressing scenarios while watching falls, particularly on anticipating negative outcomes.	<J2445>
During	Cognitions	Catastrophic thinking	Engaging in catastrophic thinking about potential injuries and outcomes of falls, imagining severe consequences.	“The first thing that comes to my head, was ok: the babe is going to fall, the rope is not going to work, and she’s going to fall into the void, and she’s going to kill herself.”	Engaging in catastrophic thoughts about potential severe injuries and fatal outcomes resulting from falls, imagining worst-case scenarios.	<J2445>
After	Emotional states	Relief	Experiencing relief when falls do not result in fatal outcomes, despite severe injuries.	“It’s like, the happy ending, like ‘aaaahh,’ he caught...It’s like the guy fell, aahh! and he killed himself, but I do not know if it’s worse, maybe, he did not kill himself, you know what I mean?”	Relief experienced when falls do not result in fatal outcomes, despite severe injuries, as it contrasts with catastrophic thoughts and imaginations of fatal outcomes.	<J2499>

### Global temporal analysis

The subsequent results demonstrate the capacity to identify a global temporal structure across all participants (Table 2):

**Table 2:** Temporal description.

Before the fall	During the fall	After the fall
Anticipation (10)		
Nervousness (10)	Nervousness (8)	
Chest/Stomach Tension (9)		
Anxiety (8)	Anxiety (12)	Anxiety (12)
Accelerated Heart Rate (3)		
Fear (5)	Fear (8)	
	Increased Tension (13)	
	Astonishment (1)	
	Rage (2)	
	Frustration (1)	
		Decreased Tension (13)
		Relief (6)
		Worry (5)
		Displeasure (5)
		Entertainment (1)
		Disturbance (1)

“Before the fall:

*Anticipation is a constant in this stage. The interviewees experienced a wide range of emotions and bodily reactions, with anxiety, nervousness, and tension in different parts of the body, especially in the chest and stomach, predominating. Some also reported more physical symptoms, such as accelerated heart rate, chest tightness, and altered breathing. Other feelings expressed include fear, stress, restlessness, concern, and expectation.*

*During the fall*:

*Tension and anxiety generally intensified during the fall.* Var*ious intense bodily responses were reported, including muscle tension, altered breathing, chest tightness, and in some cases, nausea and physical imbalance. The predominant emotion remains fear and nervousness, though some individuals also experienced astonishment, rage, and frustration. At this point, some people also described feelings of displeasure and anger, especially in response to falls they perceived as avoidable or irresponsible.*

*After the fall*:

*After the fall, most interviewees experienced a decrease in tension and anxiety, although in many cases, these sensations persisted to some degree. Reactions ranged from relief and entertainment, to concern, discomfort, displeasure, and disturbance. Some interviewees also expressed the need to reflect on what they had seen, with a focus on safety and the potential consequences of falls.*

*In summary, the experience of watching falls was emotionally intense and physically evocative for most interviewees, with a clear progression of increasing anticipation, intensifying responses during the fall, and a subsequent period of reflection and gradual diminution of initial reactions. However, there was* var*iability in individual responses, reflecting different ways of reacting to stressful and potentially dangerous situations.”*

The following table illustrates the capability to identify individual patterns and construct an overarching analysis incorporating specific experiential dimensions that delineate each phase, along with the ability to quantify them.

### Global transversal analysis

The following table succinctly summarizes the results, emphasizing the proficiency of ChatGPT in pinpointing a multitude of experiential categories and providing appropriate descriptions within the framework of the video viewing experience (Table 3).

**Table 3:** Experiential dimensions.

Category	Subcategory	Conceptual definition
Physical and bodily sensations	Muscle tension	The contraction and stiffness of muscles experienced by participants while watching the videos
	Heart palpitations and acceleration	An increase in heart rate or perception of palpitations experienced by participants while watching the videos
	Sweating and cold hands	The production of sweat and the sensation of cold hands experienced by participants while watching the videos
Emotional states	Fear and distress	The negative emotions of fear and distress experienced by participants while watching the videos of falls
	Dislike and rejection	The emotions of dislike and rejection experienced by participants while watching the videos of falls, associated with aversion toward the images
Intentions and desires	Self-protection	The desire to protect oneself from the risk situations presented in the videos
	Helping and protecting others	The desire to help or protect the people in the videos from the risk situations
Attentional focus	Focusing on specific details	Participants focus on specific details of the videos, such as body position, environment, or actions leading to the fall
	Distraction and avoidance	Participants experience distraction or avoid watching the videos due to the emotions and sensations they generate
	Concern for the people involved	Participants focus on the well-being and safety of the people involved in the falls
Reflections and learnings	Importance of safety and accident prevention	Participants recognize the importance of safety and accident prevention by observing the consequences of falls in the videos
	Empathy and solidarity toward others	Participants develop empathy and solidarity toward the people involved in the videos, understanding their suffering and difficulties

### Experiential structures

**Figure 6:**
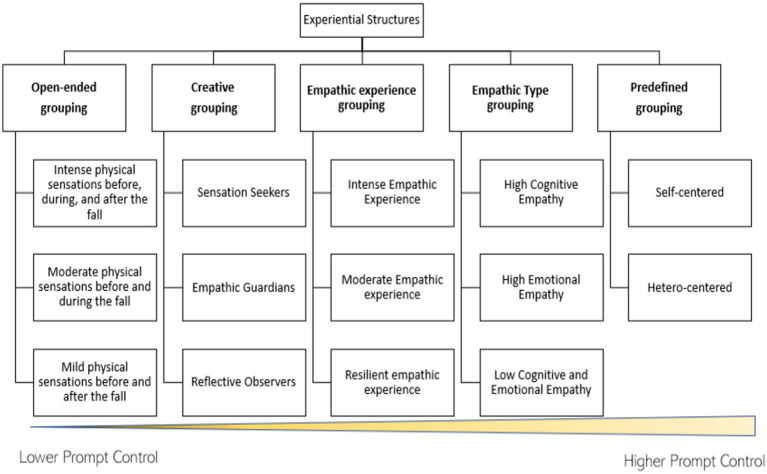
Illustrates the five distinct methods employed to investigate chatGPT’s capability to identify experiential structures and the corresponding outcomes. We employed prompts ranging from lower control to higher control in order to conduct these analyses.

The outcomes of Step 6 were depicted in Figure 6, showcasing versatility in categorizing subjects based on particular definitions. For details on the identification of the structures for each participant, refer to Supplementary Table S1.

In the following paragraphs we describe the outcomes associated with each prompt:

#### Open-ended grouping

The results obtained in this analysis are characterized by the use of experimental categories, specifically bodily sensations, to identify the structures of experiences. The results obtained from the analysis are as follows:

*“Group 1*—*intense physical sensations before, during, and after the fall (18 individuals):*

*This group is characterized by experiencing intense physical sensations before, during, and after witnessing the falls in the videos. These sensations include bodily tension, muscle tightening, accelerated breathing, a sense of imbalance, sweating, among others. Individuals in this group have a focal attention directed toward both themselves and the individuals in the videos, and they experience emotions such as fear, anxiety, concern, and empathy.*

*Group 2—moderate physical sensations before and during the fall (7 individuals):*

*This group is characterized by experiencing moderate physical sensations before and during the viewing of the falls in the videos. These sensations include muscular tension in specific areas such as legs, arms, chest, and stomach. Individuals in this group have a focal attention primarily directed toward the individuals in the videos and experience emotions such as fear, dislike, and concern.*

*Group 3—mild physical sensations before and after the fall (3 individuals):*

*This group is characterized by experiencing mild physical sensations before and after witnessing the falls in the videos. These sensations include slight muscle tension and discomfort in the stomach. Individuals in this group have a focal attention directed toward themselves and the individuals in the videos, and they experience emotions such as fear, concern, and mild disgust.*”

#### Creative grouping

The results obtained in this analysis are characterized by the use of experiential narratives, which enable a deeper understanding of the participants’ types of experiences. The results obtained from the analysis are as follows:

“*Group 1*—*sensation seekers (4 individuals)*

*This group comprises individuals who crave intense physical sensations when watching the falls. They thrive on the adrenaline rush and experience a wide range of sensations, including heightened heart rate, sweaty palms, and a surge of energy. Their attention is captivated by the individuals in the videos, and they feel a mix of fear, excitement, and awe. There are 4 adventurous souls in this group.*

*Group 2*—*empathic guardians (16 individuals)*

*In this group, individuals are highly empathetic and protective toward the people in the videos. They feel a moderate level of physical sensations, such as a tightening in their muscles and a knot in their stomachs. Their attention is divided between themselves and the individuals in the videos, and they experience emotions of concern, empathy, and a strong desire to prevent harm. This group consists of 16 compassionate individuals.*

*Group 3—reflective observers (8 individuals)*

*The individuals in this group maintain a calm demeanor while observing the falls. They experience mild physical sensations, like a subtle tenseness in their muscles and a gentle unease in their stomachs. Their attention is focused on both themselves and the people in the videos, and they exhibit emotions of contemplation, curiosity, and a detached sense of mild discomfort. This introspective group consists of 8 thoughtful individuals.”*

#### Empathic experience grouping

The findings from this analysis are distinguished by the exploration of experiential empathy in terms of varying intensity levels. This approach offers a more nuanced comprehension of the diverse empathetic experiences among participants. The specific outcomes derived from the analysis are detailed below:

*“Group 1 - intense empathic experience (16 individuals)*

*This group of subjects experienced intense reactions of anxiety, nervousness, and stress during the video viewing. They strongly identified with the individuals in the falling situations and experienced similar physical and emotional responses. Their empathic ability led them to experience a high level of discomfort and distress, especially during the falls.*

*Group 2 - moderate empathic experience (9 individuals)*

*The subjects in this group showed a moderate empathic experience. They expressed feelings of astonishment, fear, and stress, but at a less intense level than the first group. They were able to maintain a certain emotional distance during the viewing, although they did experience some degree of discomfort and tension.*

*Group 3 - low empathic experience or resilient (3 individuals)*

*Description: This subject experienced feelings of discomfort and disgust but was able to quickly recover after watching the videos. Although the initial experience was uncomfortable, the resilience of this subject allowed them to return to a state of calm and relief quickly*.”

#### Empathic type grouping

For this classification, ChatGPT considered two fundamental types of empathy: cognitive and emotional. However, a third group was identified and added based on the low intensity of both types of empathy exhibited by the participants. The results obtained from the analysis are as follows:

*“Group 1—high cognitive empathy (8 individuals)*

*This group of subjects has demonstrated a strong ability to understand and share the perspective of individuals in the videos. Their capacity to “put themselves in someone else’s shoes” is remarkable and allows them to comprehend the situations in a deep manner. However, this understanding does not always translate into intense emotional responses.*

*Group 2—high emotional empathy (18 individuals)*

*The subjects in this group are highly emotionally responsive. They feel a strong emotional connection with the individuals in the videos, leading to intense reactions of anxiety, fear, and stress. While they understand the situations, their ability to manage the intense emotions they experience may be lower.*

*Group 3—low cognitive and emotional empathy (2 individuals)*

*This subject has shown limited capacity for both cognitive and emotional empathy. Although they understand the situations and experience certain emotions, these responses are less intense and more manageable compared to the other groups.*”

#### Predefined structures

The outcomes of this analysis highlight ChatGPT’s capability to categorize the subject according to our previous definition of structures of experience. One subject was included in both groups because of their dual experience. The results obtained from the analysis are as follows:

*“Group 1—self-centered empathy (24 individuals)*

*Subjects grouped under this type of empathy tend to focus on their own reactions and feelings. These individuals tend to be more sensitive to negative emotions and may experience higher anxiety or stress when facing difficult situations. Self-centered empathy is often associated with a tendency to avoid or protect oneself from emotionally charged situations.*

*Group 2—other-centered empathy (5 individuals)*

*Subjects with this type of empathy focus more on the feelings and experiences of others. They genuinely care for people who are experiencing difficulties and feel motivated to help. This type of empathy can be very rewarding, but it can also lead to emotional burnout if not managed properly.*”

## Discussion

This paper seeks to introduce a scalable methodology for qualitative analysis that is designed to be effectively integrated with large sample sizes, enhancing the robustness and applicability. Our proposed approach utilizes ChatGPT, a Large Language Model adept at Natural Language Processing (NLP), to analyze data from in-depth phenomenological interviews. This method aims to maintain the integrity of qualitative insights while leveraging the robustness of quantitative methods, enabling a comprehensive and nuanced understanding of complex phenomena. Our methodology unfolded across four crucial stages: starting with the Preparation of Phenomenological Data, where interview transcriptions were refined to ensure relevance to the research question. This was followed by the Individual Analysis, where ChatGPT delved into each transcript to underscore critical nuances of individual experiences. The Global Analysis phase then reexamined these insights through both temporal and transversal perspectives until a comprehensive understanding was achieved. The process culminated in the Structure of the Experience phase, a synthesis step that outlined the fundamental and structural components of shared experiences. Essential to each stage, except the first, was the creation of bespoke prompts, tailored to meet the specific aims of each phase and to capture the experiential elements accurately. In our findings, ChatGPT demonstrated a high level of skill in phenomenological analysis, effectively capturing subtle aspects of human experiences. ChatGPT displayed a high level of skill in phenomenological analysis, effectively capturing subtle aspects of human experiences. Its ability to organize experiential themes—such as emotions, bodily sensations, and cognitive responses—demonstrates an advanced processing capability for nuanced human interactions and responses. By identifying these dimensions within complex data, ChatGPT provides clear and coherent insights that reflect the richness of lived experiences, illustrating its proficiency in handling intricate qualitative information. While our findings suggest that ChatGPT can successfully identify relevant phenomenological findings, we recognize the need to further explore how these AI-generated results align with those produced by human analysts. Advancing this line of inquiry through systematic cross-validation will be essential for consolidating the methodological reliability of this approach. The discussion section revisits this issue at several points, outlining both the limitations and possible solutions for developing a more rigorous and transparent methodology.

### Methodological comparison: traditional vs. AI-assisted analysis

Traditionally, qualitative analysis has relied on human coding. Yet, our innovative method paves the way for incorporating artificial intelligence in the qualitative analysis process. Although both approaches yield a rich and in-depth analysis of human experience, they fundamentally diverge in their analytical techniques. Human phenomenological analysis, historically distinct from other qualitative methods, invokes epoché and reduction, principles that facilitate a pure and impartial analysis of experiential narratives (Giorgi, 1994, 2010; Giorgi et al., 2017; Van Manen, 2017). This approach requires the analyst to suspend their judgments and preconceptions to fully immerse in the phenomenon, focusing solely on the lived experiences as described by the participants. Through a meticulous process of data immersion, coding, and thematic analysis, researchers engage deeply with the text to extract essences and meanings. At its core, phenomenological analysis is devoted to capturing the essence of lived experience (Zahavi, 2021), a principle upheld in both traditional and AI-assisted methodologies. While human analysis employs epoché and reduction to approach this essence through direct interaction and deep reflection, ChatGPT-assisted analysis adapts this fundamental goal to a technologically advanced setting. ChatGPT, with its advanced algorithms and extensive training data, can intricately parse and interpret text, discerning the underlying themes and sentiments. It operates by detecting recurring linguistic patterns and structures that signify certain experiential elements. This means it can recognize how language is used to express emotions, sensations, and thoughts, often indicative of a person’s experiences. By doing so, it can generate a descriptive analysis that may encapsulate the essence of the subjective experiences conveyed in the data it processes.

Importantly, however, it is crucial to recognize that human and AI-assisted analyses are not mutually exclusive but are complementary components of a robust mixed-methods framework. In large-scale studies, the analytical process might commence with traditional human coding to establish a deep initial understanding. Upon reaching analytical saturation, the study can seamlessly transition to AI-supported phenomenological analysis. This integration harnesses the strengths of both approaches: the nuanced understanding from human analysis and the expansive analytical capabilities of AI. Such a collaborative use of supervised learning enriches the insights derived from human expertise, extending the scope and depth of the overall research.

### Efficiency and scalability of ChatGPT analysis

The potential efficiency of ChatGPT in phenomenological analysis becomes palpable when considering the time invested in the process. To perform phenomenological analyses with this same data (Martínez-Pernía et al., 2023), our team of three researchers dedicated 70 h. In contrast, a conservative estimate of the time required for the analyses presented here is 20 h. It’s important to note that much of the time involved in this AI-assisted process was spent on crafting manual prompts, which appears to minimally impact the feasibility of handling extensive datasets. This significant reduction in analysis time not only demonstrates ChatGPT’s efficiency but also addresses several inherent challenges of human coding in qualitative research. These include the labor-intensive nature of manual coding, challenges in achieving thematic saturation, the subjective variability in narrative description, and the potential dilution of personal engagement with large data sets. For any researcher planning on conducting phenomenological or general qualitative analysis, estimating the workload is a crucial factor that determines feasibility. Thus, highlighting this significant time-saving potential stands as a major contribution of our work. Building on this, the scalability of our methodology aligns seamlessly with the goal of enhancing scalability in qualitative analysis method studies. This analysis markedly reduced the time required compared to traditional human coding, effectively addressing the critical challenge of handling large sample sizes efficiently. This aspect underscores our method’s capability to facilitate broader research applications without sacrificing the depth of qualitative inquiry.

### Comparative performance evaluation: human vs. ChatGPT analysis

Following, we compare the performance of ChatGPT-assisted phenomenological analysis with the human analysis conducted on the same set of interviews, as detailed in a prior publication (Martínez-Pernía et al., 2023). Similar to human analysis, ChatGPT offers a detailed analysis of individual bodily sensations and temporal dynamics, while also identifying important elements like self-protection and prosocial motivation in participants’ experiences. Another notable similar feature is ChatGPT’s ability for thematic organization, abstracting higher-level dimensions like motivation or bodily sensations. However, two significant improvements stand out in the AI-assisted approach. Firstly, enhanced specificity is evident where human analysis tended to describe emotional and bodily aspects more generally, ChatGPT demonstrated a superior ability to detect specific, concrete dimensions of experiences. For instance, the AI was precise in identifying nuanced physical reactions, such as sweaty or cold hands, which were not as distinctly noted in the human analysis. Secondly, ChatGPT showed exceptional flexibility in generating varied experiential groupings based on the analysis prompts. This adaptability allows researchers to explore new ways of categorizing data, potentially offering novel insights that can enrich the theoretical underpinnings of phenomenological research.

Despite these strengths, our study also noted certain limitations in the AI-assisted approach. While there is considerable overlap in the findings of ChatGPT and human analyses, complete congruence is not always achieved, particularly in identifying complex phenomenological structures such as self-centered and other-centered empathy. Additionally, a notable difference was ChatGPT’s occasional omission of kinesthetic sensations as categories, which were identified in human-coded analyses. These observations underscore the need for future cross-validation between AI-assisted and human-conducted methods. Upcoming research should aim to rigorously examine how these two approaches diverge, and what such differences may imply for the validity and reliability of phenomenological inquiry. By pursuing systematic cross-validation, we can better delineate both the potential and the limitations of integrating AI tools like ChatGPT into qualitative research, ensuring that human insight and technological innovation contribute in complementary ways to the depth and rigor of phenomenological analysis.

Beyond these comparative findings, it is also important to recognize a broader limitation in using LLMs for phenomenological analysis. While these models may help reduce certain forms of bias by offering standardized summaries, they also introduce new ones. Since LLMs are trained on large text corpora shaped by public discourse, they may produce what could be called “dual experiential structures”: descriptions that fit the way we usually speak or write, but that do not necessarily match the actual structure of lived experience. As Gallagher and Zahavi (2020) note, not everything we experience is immediately available in language, and there is a risk of mistaking what can be easily verbalized for what was genuinely experienced. This calls for careful interpretation when working with AI-generated descriptions of subjective phenomena, and reinforces the importance of both future cross-validation studies comparing AI and human-led analyses, and the development of human-guided strategies—such as model fine-tuning or structured APIs—to mitigate these limitations.

### Integrative applications of ChatGPT in mixed-method studies

One of the primary applications of this methodology is in large-scale qualitative studies, where it facilitates the in-depth analysis of extensive datasets. Additionally, it proves invaluable in integrative mixed-method research, designed to merge quantitative and qualitative findings. A notable example of its application is the ‘middle way’ research approach, which seeks to unify the neurobiological aspects of consciousness with phenomenological attributes (Varela, 1996; Colombetti, 2013; Depraz and Desmidt, 2019; Froese, 2015; Troncoso et al., 2023). The emergence of automated methods for phenomenological analysis, such as the potential demonstrated by ChatGPT, signals a promising path for these research models to conduct comprehensive investigations of consciousness, integrating its diverse attributes in a holistic manner. Within the context of ChatGPT-assisted phenomenological analysis, it becomes imperative to illuminate how this innovative approach resonates with the rigor of quantitative methods prevalent in cognitive sciences. For instance, ChatGPT-assisted phenomenological analysis offers the possibility of advancing toward a process of mutual co-determination and co-validation in cognitive sciences. The validity of the results stems from the dynamic interplay between two dimensions of consciousness: experience and neurobiology (Petitmengin and Bitbol, 2009). This approach entails more than merely correlating neural structures with experiences; it aims to foster and facilitate a process where each informs and substantiates the other. Moreover, ChatGPT-assisted analysis enhances the study with a degree of near-intersubjectivity. It acknowledges the complexities involved in describing experiences while closely associating them with their neurological bases. Through this approach, ChatGPT-assisted analysis does more than just describe; it actively contributes to the validation of subjective experiences, anchoring them in intersubjective agreements.

### Ethical challenges

The LLMs like ChatGPT in experimental contexts raise significant ethical challenges that demand careful attention and critical reflection. While we implemented methodological measures aimed at mitigating risks associated with data privacy, misinterpretation of information, and the introduction of biases (see Supplementary material), ethical concerns remain that require deeper consideration. One of the primary ethical challenges is ensuring the privacy and confidentiality of participants. In this study, although we applied a rigorous process of anonymization and data cleaning to conceal Personally Identifiable Information (PII) before submitting interviews to ChatGPT, the model’s opaque nature—essentially operating as a “black box”—raises questions about the absolute effectiveness of these measures. This issue has been identified as a critical area that requires stronger technological developments and specific mechanisms to ensure the continuous security and protection of confidential information (Khowaja et al., 2024). In relation to this issue, it is important to clarify that in this study, all interactions with ChatGPT were conducted through the user interface rather than via the API. According to OpenAI’s policies, data provided through the user interface is not used for training purposes (https://openai.com/es-ES/policies/row-terms-of-use/). Therefore, the interviews we processed are secure and were not accessible for training or model improvement by OpenAI. This measure was taken precisely to enhance the protection of sensitive information and minimize risks of unauthorized access.

Another critical ethical issue concerns bias management within AI-generated outputs. It is well-documented that LLMs like ChatGPT are susceptible to biases embedded within their training data, which may influence their responses to prompts (Ray, 2023). Biases related to race, gender, cultural identification, and other aspects of human experience pose significant threats to the validity of AI-assisted qualitative research. In response to this concern, our methodology incorporated several safeguards aimed at minimizing biases throughout the analytical process. These measures—detailed in the Supplementary materials—included careful data cleaning, participant anonymization, the use of structured prompts that required the model to base its responses on direct quotations, randomization of interview subsets to avoid order effects, and continuous human supervision of the analysis. Despite these safeguards, this challenge may still persist. Subtle forms of bias can emerge from the complex and often opaque interactions between the AI model and the data, and human supervision—while essential—is not exempt from cognitive limitations. While we have implemented rigorous measures to reduce bias and protect participant privacy, the use of LLMs such as ChatGPT continues to pose ethical complexities that demand ongoing critical reflection and methodological refinement. Strengthening collaboration between researchers and developers is essential to advance the development of monitoring systems and analytical tools that ensure more robust and ethically grounded research practices (Pathak, 2023; Zack et al., 2024).

### Implications for future research

We acknowledge that the current work depends critically on our ability to craft prompts. However, models can be supplemented with information about how to handle specific topics to gain fuller advantages of LLMs capabilities. We envision future work where a pre-trained transformer model is fine-tuned with personalized human feedback according to the goals of the research team. However, this is a costly and time consuming alternative, requiring a thoughtful consideration of the cost to benefit ratio. A cheaper alternative is to develop a customized API. Given that in the current work we have been successful using prompts, an API could be built with a set of instructions on how to handle requests from the phenomenological analyzer, limited only by the ChatGPT’s maximum number of tokens requirements. These instructions are messages to the system, which set the context for interpreting users’ requests. For example, if researchers are interested in the experience of *openness*, the system instructions would contain a definition of that concept, to be used when answering requests (for details about these and other alternatives, please see https://platform.openai.com/docs/api-reference/fine-tuning/retrieve).

A central challenge that emerges from AI-assisted qualitative analysis is reproducibility and replicability—issues that have long persisted in psychological and neuroscientific research (Button et al., 2013; Marek et al., 2022; Simmons et al., 2011). While we designed prompt structures to maximize internal consistency across analyses, we acknowledge that different datasets—or even different versions or instantiations of LLMs—may yield variable outputs. This limits the direct replicability of our exact procedure. Although the structure and logic of the prompts can be reused, fine-tuning may be necessary for other datasets or research questions. A key limitation of this methodology is that ChatGPT’s proprietary nature complicates transparent examination of its inner workings, making it difficult to replicate analysis pipelines across contexts or over time (Bender et al., 2021). One promising avenue for addressing this issue involves the development of open-source AI agents—large language models whose code, architecture, and parameters are publicly available—for integrative research. Open frameworks not only provide inspectability and auditability of algorithms but also empower the broader research community to retrain, fine-tune, or extend the models under equivalent conditions, thereby enhancing methodological rigor (Wolf et al., 2020). Furthermore, open-source initiatives can facilitate model version control, enabling researchers to report precisely which iteration of the model was used—a key factor for reproducibility in rapidly evolving fields (Peng, 2011; Stodden et al., 2014). By embracing these open-source developments, future studies can strengthen the credibility of ChatGPT-assisted phenomenological methods, ensuring that both the depth of qualitative insights and the robustness of quantitative approaches remain firmly anchored in transparent and verifiable practices.

## Conclusion

In conclusion, the present research underscores the capabilities of LLMs such as ChatGPT in elucidating intricate experiential narratives. Drawing from phenomenological interviews, this study effectively leveraged ChatGPT to transform raw, loosely structured input into rich and detailed phenomenological accounts. The systematized output from our AI-assisted qualitative method, driven by meticulously designed prompts, not only clarifies complex human experiences for a deeper conceptual understanding but also highlights the sophisticated analytical advantages of employing large language models. While there remains scope for enhancement, the method’s profound capacity for understanding and categorizing data underscores its significant potential as a qualitative research tool. Unlike traditional methods that rely solely on human coders, our findings demonstrate that ChatGPT can offer thorough insight with significant cost and time efficiencies. This innovative approach does not aim to supplant human coding but rather to complement and integrate with it, thereby enriching the analytical process. Consequently, this methodology is poised to expand the scope of qualitative research, providing a robust and scalable framework that marries the depth of qualitative insights with the systematic strengths of quantitative analysis. Through this integration, we forge a path forward for qualitative research that accommodates large data sets without sacrificing the granularity of human experience, setting a new standard for depth and efficiency in qualitative analysis.

## Data Availability

The original contributions presented in the study are included in the article/Supplementary material, further inquiries can be directed to the corresponding author/s.
