# Supplementary material for: Bridging consciousness and AI: ChatGPT-assisted phenomenological analysis
Source: Front Psychol. 2025 May 29;16:1520186. doi: 10.3389/fpsyg.2025.1520186 (PMC12159045; doi:10.3389/fpsyg.2025.1520186)
Supplement: Supplementary file 1 [file Data_Sheet_1.pdf]

Supplementary Material

1. Description of Interview

All interviews were performed in Spanish by the same investigator (DMP), captured via audio recording, and transcribed word-for-word. To initiate each session, participants were prompted to describe videos they perceived as unpleasant and identify the one that evoked the strongest reaction. This technique was designed to enhance the clarity of their memories related to the experience. Descriptions during the interview focused solely on the participant’s reactions to their selected video, inspired by the micro-phenomenological interview method (MPI) (Petitmengin, 2006).

The interview method explored the multidimensional aspects of the participants' experiences (such as bodily, emotional, sensory, and attentional responses) and tracked how these elements varied over time. The interview began with an explanation of its goals and the methodology's emphasis on an embodied approach to querying and responding, centered on the video-related experiences. Participants were then encouraged to vividly recall their video experience, which is essential for eliciting their pre-reflective descriptions and thoroughly examining their prior experiences (Petitmengin et al., 2019). The conversation focused on both the synchronic (simultaneous) and diachronic (over time) aspects of the experience, with questions like "How do you feel about the video?" or "What is the feeling of tension like?" and follow-ups such as "After feeling tension, what occurs?" or "At what moment do you feel the tension?" A key part of the interview involved summarizing the participants’ responses to aid their recollection of the experience.

Here, there is a description of the common structure and examples of interviews.

a) Initial instruction:

"Please, if you feel comfortable, close your eyes and take a moment to connect with your experience of being on the platform and watching the videos of the people who fell. Remember it as if you are living it right now, please let me know when you are ready."

b) Examples of the questions asked in the interview, according to the experience:

1. How did you feel at the beginning of the video?
2. How did you feel when the person fell?
3. Was it before or after [that]?
4. What happened next?
5. What did you feel when [that happened]?
6. How did your body feel?
7. What sensations did you feel?
8. What were those sensations like?
9. Did you feel that sensation in any particular place in your body?
10. How did you know you were feeling that way?
11. You mentioned a sense to avoid watching the videos.
12. How was that like?
13. At that moment, what did you want to do?
14. Was what you just told me accompanied by a thought?
15. How did that thought arise?
16. Did you imagine it, did an image come to you or how did it appear?

As the interviewer goes through the different dimensions, he/she recapitulates the experience as the subject makes it known. In this way, the interviewer tries to constantly return the participant to the experience itself so as not to lose focus.

At the end, the participant is asked if there is anything about the experience that has not been asked or that he/she would like to add and that he/she considers significant for the understanding of his/her experience.

2. Ethical Considerations

The implementation of LLMs such as ChatGPT, raises several ethical challenges and caveats for empirical research that are not fully understood (Van Dis et al., 2023). For that reason, it is imperative to identify potential ethical concerns as a measure to mitigate or avoid unethical behavior in research. Below we summarize some ethical conflicts that were identified alongside the present work and how we address them.

Data privacy and confidentiality: As it is shown in Step 1 of data preparation, all personal data of each participant were properly anonymized (i.e., replacing participant's names with acronyms). Additionally, a data cleaning process was conducted, in which any non-relevant information (e.g., personal information) from the interviews were removed.

Misinterpretation and misrepresentation of data: Recently, a phenomenon known as hallucination has invaded LLMs research (Ji et al., 2023). Hallucination occurs when a language model inadvertently provides an inaccurate response to a given prompt. Moreover some studies suggest that ChatGPT may inadvertently generate a chain of incorrect responses, known as a snowball effect. Given the model's reliance on prior responses, it may perpetuate errors in subsequent responses based on incorrect feedback (Zhang et al., 2023). To mitigate this challenge, specific rigor criteria were introduced during the analysis to ensure that our conclusions remained closely tied to the original interviews. For example, tables were generated to link categories, definitions, quotations, and their specific locations within the interview.

Data reliability: A major concern in relying on ChatGPT (and any other LLMs) is that their training sets are generally not openly available to the public. Thus, it is unknown to the user how ChatGPT is generating its outputs. To that extend, ChatGPT seems to operate as a "black box" in where the user does not know how the model solves a given problem (in the form of a prompt) (Aiyappa et al., 2023; Chen et al., 2023). One alternative to address this issue is to create specific validity criteria for the prompts designed to avoid potential inconsistencies. For instance, we proposed a standard prompt operationalization to ensure inner prompt consistency across individual interviews (Steps 2 and 3) and across interview groups (Steps 4 and 5) (Figure 1). The main goal was to provide clarity, consistency, and comparability in the analyses through a diverse interview context.

Bias: To mitigate the possible bias in the process of the analysis, we implemented several methodological procedures. Firstly, we employed rigorous data-cleaning procedures to eliminate extraneous influences from the interviews, thereby reducing the potential for bias

introduced by irrelevant content (Creswell & Clark, 2017). Additionally, participant information was anonymized to mitigate identification bias and promote data integrity (Saunders et al., 2015). Secondly, we designed structured prompts that ensured the AI analysis remained closely aligned with the original transcripts. These prompts explicitly instructed ChatGPT to include references and direct quotes from the interviews, thereby minimising interpretative drift and hallucinations (Ji et al., 2023; Zhang et al., 2023). Thirdly, we randomized the interview subsets to reduce ordering effects and avoid biases stemming from the model's reliance on prior prompts (Morse & Niehaus, 2016). This approach contributed to a more robust analysis by promoting consistency and fairness across different data subsets. Furthermore, the AI-generated categorizations were carefully cross-validated and supervised by human researchers. This dual approach aimed to enhance the reliability and phenomenological rigor of the analysis, recognizing that human supervision is not exempt from cognitive biases (Giorgi, Giorgi, & Morley, 2017; Van Manen, 2017). Together, these measures helped to establish a transparent, consistent, and methodologically rigorous analytical framework.

3. Example of Interview

Below we have left interview 24, which was used as an example in the analysis of the individual phases. We have left one translated to English from the original language (Spanish) and the original one.

3.1 Translated into English

<J241> I: What did the videos consist of?

<J242> E: Eeee, horrible, I, I panic the. First, well, I said I panic falling [em, em], I get that feeling of... I've had so many sprains in my ankle, and the pain is so intense that it's more, when I see someone fall, and they bend in the joint, it hurts me [em, em]. It even makes me nauseous; no, no, I cannot. When I see the videos, I saw the videos that were much stronger [smiles] than a sprain, eeee, the feeling that they are going to, I mean, I don't know, it says that their head will burst, that they will break their spine, a cervical spine, that they will not be able to walk anymore, that... it was worse... I mean, their hands are no longer sweaty, they were all wet, I don't know, eeee. The fact that they fall badly, that, that they fall on their face, that, that they do not manage to support their hands, or that, because of course! they don't end up seeing the fall, that is, at least in the one that is climbing the wall, they don't end up seeing the fall well, or I, again, no, I was so, so hysterical that, I don't know, I don't feel that I

can support my hands, that you, that you can see an exposed fracture, that aaaah! I: have any, any of the videos of the falls given you a positive sensation?

<J243> E: No. [very low].

<J244> I: No. Any, neutral feeling?

<J245> E: No. [I understand].

<J246> I: No. Did the six videos give you a negative feeling?

<J247> E: Yes, the worst one, the one with the wall.

<J248> I: The one on the wall, which one is he? the guy who is

<J241> I: What did the videos consist of?

<J242> E: Eeee, horrible, I, I panic the. First, well, I said I panic falling [em, em], I get that feeling of... I've had so many sprains in my ankle, and the pain is so intense that it's more, when I see someone fall, and they bend in the joint, it hurts me [em, em]. It even makes me nauseous; no, no, I cannot. When I see the videos, I saw the videos that were much stronger [smiles] than a sprain, eeee, the feeling that they are going to, I mean, I don't know, it says that their head will burst, that they will break their spine, a cervical spine, that they will not be able to walk anymore, that... it was worse... I mean, their hands are no longer sweaty, they were all wet, I don't know, eeee. The fact that they fall badly, that, that they fall on their face, that, that they do not manage to support their hands, or that, because of course! they don't end up seeing the fall, that is, at least in the one that is climbing the wall, they don't end up seeing the fall well, or I, again, no, I was so, so hysterical that, I don't know, I don't feel that I can support my hands, that you, that you can see an exposed fracture, that aaaah!

<J242>I: have any, any of the videos of the falls given you a positive sensation?

<J243> E: No. [very low].

<J244> I: No. Any, neutral feeling?

<J245> E: No. [I understand].

<J246> I: No. Did the six videos give you a negative feeling?

<J247> E: Yes, the worst one, the one with the wall.

<J248> I: The one on the wall, which one is he? the guy who is

<J249> E: [The boy who goes climbing.

<J2410> I: The most intense one? The boy who climbs, right?

<J2411> E: = Yes.

<J2412> I: What is the second most intense?

<J2413> E: = Aaam, wait for it, there were several, they were going like this, like, like on a skateboard,....

<J2414> I: Yes, there was one....

<J2415> E: [But their falls were very similar: the skateboard came out that way; the other one, this way.

<J2416> I: There was one that was driven by a pickup truck....

<J2417> E: [The one of::, no. The one of the, the one of the snow.

<J2418> I: Ok. There were three snow: one, two

<J2419> E: The one who fell into the railing with red mesh.

<J2420> I: Ok.

<J2421> E: The one who

<J2422> I: The first one?

<J2423> E:I: So, in, the one with the most intensity is the climbing one?

<J2424> E: Climbing and then the de...

<J2425> I: [Then

<J2426> E: = Skiing.

<J2427> I: And the others from skiing would also follow you?

<J2428> E: Yes, more than skateboard falls because....

<J2429> I: Ok. Ok.

<J2430> E: = Because I find it, I don't know,

<J2431> I: And the, and the other one that would be left, is just the one of the girl who is going up a rope at, at very high altitude....

<J2432> E: [Ah! no, no, no, no, no, no, you're absolutely right. It's just that I think it's stupid, even more so with heels.

<J2433> I: Yes.

<J2434> E: Look, the first thing I noticed, when I saw the video, was that she had a, like a bicorné, [em] to tie herself in case she fell, but the first thing I imagined is that if the girl fell, the harness was going to cut [ok], and she was going to fall.

<J2435> I: And that one that....

<J2436> E: = I never imagined that it would be fastened, never.

<J2437> I: Ok. And that one in what, what graduation do you give it within the videos: the first one, the second one, the third one,... in intensity of distress?

<J2438> E: In intensity of distress, I, I had missed it: that's the one; the two, the scale; and the three, the snow.[smiles].

<J2439> I: I mean, the first one is the....

<J2440> E: [I had completely forgotten about it.

<J2441> I: [The girl's,

<J2442> E: = Yes.

<J2443> I: Ya. Ok.

<J2444> E: I think what I blocked it. [laughs] I panic the, I now, as a grown-up, I panic heights [em, em]. I used to love heights, I like games, extreme games, like roller coasters and everything, but heights, leaning out from a balcony, I prefer to be, boy! leaning out from behind the balcony [em, em], I'm not interested, and when I see him who is very close, I feel that his body could go to the balcony, no! I don't go near balconies [ok]. I can't. How do you perceive, how does your body respond to the exposure of the video?

<J2445> E: No, first, eeee, not wanting to watch it: didn't want to watch any video, eeee, hand sweating, much more than the previous ones hey!eeee, I wanted to turn off the TV [smiles] [em, em]; I didn't want to watch, I just didn't want to watch [em, em], but the sweating of hands was not even an anguish in front [short pause] because it was different; I don't know: they were falls [em, em], eeee, and there were no other people involved, it was the person who was doing it because he wanted to, not because he was forced to [em, em]. Eeee, no, it's a, what it is that I didn't want to look at it, no, no, I had no desire to look at it [em ,em] [ok] because no, as I tell you, the feeling of the girl on the tightrope with heels, my first image, the first thing that comes to my head, was ok: the babe is going to fall, the rope is not going to work, and she's going to fall into the void, and she's going to kill herself. To me, that was the logic. I didn't even want to state or that I was going to have her dangling like a movie, ouch, that I was going to go rescue her or not. I didn't even get past that. As far as the, of the, of the ones on the wall that he's climbing, eeee, the first thing I find it stupid, [em, em] but stupid because of the stupidity, not that, because of the stupidity that he does, the risk that he takes, that's what I mean [em, em]. Eeee, it's that he falls on concrete [em, em], I mean, some mats don't give me that feeling, the same,... but falling on concrete concrete seems [em, em] painful, I don't know [when]. I'm sorry you didn't break into a thousand pieces

<J2446> I: ok?

<J2447> E: [There's also I was blocked, that I couldn't move. No, it was, I didn't even think about it. It's like I don't know, ouch, that same feeling.

<J2448> I: [I mean there was about a

<J2449> E: [Rejection. Yes, to a rejection.

<J2450> I: Knowing that you had to not move, did you move?

<J2451> E: =At that moment, I forgot that I couldn't move [silence]. I remembered that I couldn't move my arms [em, em], [smiles] but I didn't remember my head [em, em], and I needed, because of course! I can do like this, but it's not the same to do like this; it's like, it's like [smiles] a self-protection, I don't know [ok].

<J2452> I: So a physical sensation that re, that [yes] you felt, was....

<J2453> E: [It was innate.

<J2454> I: [It was to close my eyes and

<J2455> E: [Shake head

<J2456> I: Let's see if with that little exercise of objectivity, you manage to recover a little more bodily [expensive] sensations.

<J2457> E: = Face, muscles of the face.

<J2458> I: What's wrong with them?

<J2459> E: (ttt) Tensos.

<J2460> I: Ya.

<J2461> E: I clenched them a lot: the teeth, the jaw [em, em], eeee, I clenched them a lot, in fact it was marked even the teeth in my mouth [em, em] and::::, but it was more, it was more... this time it was like more of neck, tense neck, face, the muscles all clenched; eeee. the hands, I don't remember if I held them, eeee, because they were, eee, sweaty, but I must have held them, I think as a reaction moment when I closed my eyes, when I didn't want to look at what was happening.

<J2462> I: Did you feel, did you feel tension in your hands?

<J2463> E: = Yes. [ten] All the time.

<J2464> I: [Voltage to close or voltage to open?

<J2465> E: No, it was like this, like waving your hands when something [ok] happened, when something was going to happen.

<J2466> I: In some oca, the hands, I understand it's the five little fingers with the palm, any other part of the, of the arm?

<J2467> E: [brief pause] Eeee, the shoulders.

<J2468> I: Shoulders?

<J2469> E: = Yes.

<J2470> I: What's wrong with the shoulders?

<J2471> E: I shrunk them.

<J2472> I: = Do they shrink?

<J2473> E: = Yes.

<J2474> I: Ok. Any more muscular feelings?

<J2475> E: Eeee, it's not that I felt tense all over: my legs, my back, my neck; I mean, I say face and neck because the face, of course! I tightened it more because it was, because I was gesticulating more [em,em], and the neck, when I moved it, I also tensed all over with my hands, but as an innate reaction of what I was looking at [already], but of course! my stomach was tight....

<J2476> I: [I mean, [yes] is it a feeling of having the whole body tense?

<J2477> E: = Yes.

<J2478> I: Ya, this muscle tension that you feel generalized [em, em], does it graduate throughout the videos, do you feel like it fluctuates this tension, or is it constant?

<J2479> E: Aaam, no, it happened to me more with the two videos, with the ones of, eeee, the climbing, with the girl on the rope. With the others, it wasn't so much, especially with the skateboarding ones: no, there, it kind of lowers my tension, it kind of goes away... [ok, ok].

<J2480> I: I mean, was there like two videos specifically [yes] where you felt like it was more muscle tension?

<J2481> E: = Yes.

<J2482> I: What, stay with these two videos, where you say that the muscle tension is maximal; and now tell me if, throughout these two videos, in the same video sequence, a fluctuation of muscle tension appears as the video progresses.

<J2483> E: [short pause] Eeee, [short pause] in the climbing one, I name it for two (septa) [yes], each one: the climbing one, when he gets to the top, and when he already realizes that he's not going to, that he can't reach the leg to [em, em] support himself. There, there was my maximum tension [ok]. And the girl...

<J2484> I: [And do you feel that when once it hits, does it go away, does it go down, or does the tension continue at the same level?

<J2485> E: Eeee, no there I feel like out, [smiles] like not ( ) [well], eeee.

<J2486> I: But the tension?

<J2487> E: The tension [brief pause], I think it decreases, but about 20%, no, not more than that.

<J2488> I: Ok.

<J2489> E: ['s throat clearing] And the girl, I was tense all the time [em, em]. And on top of that looking at her from underneath, I would have seen her with sneakers, the tension wouldn't have been so much; as I saw her with heels, and for me it was worse: it was more tense the situation with the heels [em, em]. Eeee, [pause] I felt that, [throat clearing] when I was like expectant, at the moment when she was going to fall because I already knew [em, em] that she was going to fall [em, em]. So, I was expecting at what point [clawing] she was going to slip or lose her balance and it was like, com:[joy] affirmative! Yeah, like, like, like the urge to [em, em], but [pause] I know I know I had to put them out, but it's like when, it's like, I felt like I would have felt herself like, I felt like her adrenaline [em, em] like she would have gotten all up when I saw you, when she had a roller coaster, same thing [ok]. It's like, like I felt the adrenaline of the girl at the time when it was filmed [ok]. And, when it's affirmed, my palms got a lot sweatier [ok].

<J2490> I: Here there is also, but this sensation is no longer similar to muscle tension, from what I understand isn't it, is it a different sensation?

<J2491> E: Eeee, no, no, no, it's not a sensa, it's a different sensation, it's not the same as the other.

<J2492> I: Yes, in this one, is there also a generalized muscular sensation of tension?

<J2493> E: Less than the, the, the, the one on the boy's scale [ok].

<J2494> I: But there is this [yes] distinct feeling [yes] of, like adrenaline you tell me?

<J2495> E: Yes, there is an adrenaline of, of, of danger because there you fall into a void [em, em]. The other one was closer to the ground, [em, em] so to speak it was less meters [em, em]. This was many meters high [em, em]. So, of course, you might fall from a tree or, but the sensation of getting [em, em], that adrenaline was like the one that got to me [ok].

<J2496> I: Right. Perfect. Notice, I'm going to give you a, a, a, a, a tic of what I've been, as an observer of your experience, what I've seen while you were watching the, the video, and that could correspond to a physical sensation; and I've seen you that, when you were watching the video, at some moments, you were going, "aaahhh!" [aaahhh], and there was like a sensation of taking the air and releasing it [yes] quickly. So, now that...

<J2497> E: [What anguish! Yes, what (incomprehensible words) the moment that I am living so much the video, that you don't manage to perceive everything that your body is doing [em, em] because of course! on the one hand, you have to concentrate on not moving; on the other hand, you have to pay attention to the video [already]; on the other hand, you have to, eeee, see that, I don't know, that your, that your reactions are not, that is, or are as natural as possible without moving [em, em]; then you get all the pain [X laughter] (incomprehensible words).

<J2498> I: And does this, this, this experience of, breathing [yes], like [sucking] air in sound familiar to you? Can you explain to me a little bit how, how did you feel it?

<J2499> E: It's like, let's see how I say it, it's like, like the happy ending, like "aaaahh", he caught [ok]. there, it's like he signed [ok]. It's like the guy fell, aahh! and he killed himself [ok], but I don't know if it's worse, maybe, he didn't kill himself [ok] you know what I mean?

<J24100> I: That is, you feel that this sensation, this [my hair stands up], that this physical sensation, you felt that it occurred at the moment that the relief appeared.

<J24101> E: Yes.

<J24102> I: So corresponds that (words superimposed).

E: Yes, it is the relief to the one who did not die [ok], but if he can remain, he can remain quadriplegic, paraplegic, what do I know! but not

4. Additional examples of interview summaries

Subject 01

Before the fall: the interviewee experiences a more rational than physical sensation, anticipating that the person in the video will probably fall and get hurt. They feel some nervousness, but not at the same level as if they were witnessing the event in real life. During the fall: the interviewee feels anxiety and a tightening in the stomach. They also lean back in a physical gesture of surprise and close their eyes to avoid seeing the moment of impact. These reactions indicate an attempt to protect themselves and divert attention from the disturbing scene.

After the fall: the interviewee mentions that the most disturbing videos for them were the one of the subject climbing between two pillars and the one of a woman balancing on a rope.

However, they also mention that other videos, especially those related to skiing and snowboarding, made them laugh.
Regarding their behavior while watching the video, the interviewee states that they moved more than they stood still while on the platform. In general, their experience with the videos varied from anticipation and anxiety to laughter and entertainment, depending on the specific content of each video.

Subject 02
Before the fall: The interviewee mentions that while watching the video, they experience nervousness and laughter. This laughter is accompanied by anticipation, knowing that the people in the video are going to fall. They also mention feeling tense, especially in the legs and stomach, while watching the video and waiting for the fall to happen.
During the fall: While the falls are happening, the interviewee describes a mix of emotions: laughter, tension, and nervousness. In some of the falls, they experience more tension and concern, especially in the case of the climber and the skater with a yellow helmet. In other cases, like the skiing videos, they feel more laughter and relaxation.
After the fall: The interviewee mentions that after each fall, their anticipation increases regarding the severity of the following falls. Overall, they do not pay much attention to what is happening but do experience a kind of expectation about what will occur in each video.
The interview reveals that the interviewee experiences different emotional states, bodily sensations, and intentions while watching the video. The attention focuses on the falls and their anticipation of what will happen next. The interviewee also mentions having memories of their own falls, which could influence their reactions and emotions while watching the video.

Subject 03
Before the fall: The interviewee describes that while watching the video, where people were performing dangerous actions, they felt desperate and thought it was obvious that they were going to fall. They mention their body was swaying on its own, although they don't know exactly why. They experienced a feeling of anticipation and expectation, wondering how and when they would fall, which may have accelerated their heartbeat.
During the fall: The interviewee states that, at the moment of the fall, they experienced fear, thinking about the consequences and the pain the people might suffer. They also felt anger, wondering why someone would do something so dangerous. Even though they anticipated the fall, when it happened, they were surprised and said things like "That must hurt!" or "How stupid!"
After the fall: After the falls, the interviewee continues to feel fear and concern about the consequences the fallen individuals might face. They also feel anger and question why

someone would perform such dangerous actions. Reflecting on their own emotions and reactions to the video, the interviewee also realizes that they would never consider doing something like that.
In summary, the interviewee experiences a mix of emotions and reactions before, during, and after the falls in the video. Anticipation and fear play an important role in their experience, as well as anger and concern about the consequences. These emotions and sensations seem to be present at different temporal phases concerning the falls in the video.

Subject 04

Before the fall: The interviewee mentions feeling tension and fear when watching falls in extreme sports, anticipating what the athlete should do to avoid falling. The tension manifests in their hands and stomach, although they are not consciously aware of it.
During the fall: When the interviewee sees someone in the video about to fall, they anticipate what will happen and feel physical tension in their hands and stomach. This tension is an automatic process reflected in their body and may be related to the empathy they feel for the athlete in the image. The tension is especially intense in the skiing videos.
After the fall: The interviewee acknowledges that some of the videos left them worried about the well-being of the athlete who fell, but not all of them caused the same level of disturbance or discomfort. Some videos didn't affect them as much, and one of them even seemed not that serious. However, overall, the videos generated some discomfort and concern.

Subject 05

Before the fall in the video, the interviewee experienced anxiety and nervousness, manifested in a racing heart and restlessness. Their attention was directed toward the people in risky situations in the video. Their intentions were to protect and warn the people, while their thoughts included memories of similar situations in everyday life.
During the fall in the video, the interviewee's tension and anxiety increased, especially in the last video with the girl falling from a considerable height. The anxiety was expressed through movement, such as activating the left arm and bodily instability. They also felt tension in the left forearm and hand. The interviewee couldn't do anything to prevent the fall, which caused frustration.
After the fall in the video, the interviewee's tension and anxiety decreased, as there was nothing they could do to change the situation. However, they still found watching the falls unpleasant and would prefer to avoid similar situations in the future. No sensations in the stomach were reported, but the racing heart, instability, and tension in the left arm were the most noticeable physical symptoms experienced while watching the video.

5. Subject Classification Based on Experiential Structure

The following table presents the identification of subjects based on their experiential structure.

Table S1. Subject Classification

ID	Open ended	Creative	Empathic Type	Predefined Structure	Empathic Experience
JOV_01	Mild Physical Sensations	Sensation Seekers	High Cognitive Empathy	Self-Centered Empathy	Moderate Empathic Experience
JOV_02	Moderate Physical Sensations	Sensation Seekers	High Emotional Empathy	Self-Centered Empathy	Moderate Empathic Experience
JOV_03	Intense Physical Sensations	Sensation Seekers	High Emotional Empathy	Self-Centered Empathy	Intense Empathic Experience
JOV_04	Moderate Physical Sensations	Empathic Guardians	High Emotional Empathy	Other-Centered Empathy	Moderate Empathic Experience
JOV_05	Intense Physical Sensations	Empathic Guardians	High Emotional Empathy	Self-Centered Empathy	Low Empathic Experience
JOV_06	Intense Physical Sensations	Sensation Seekers	High Emotional Empathy	Self-Centered Empathy	Moderate Empathic Experience
JOV_08	Moderate Physical Sensations	Empathic Guardians	High Emotional Empathy	Self-Centered Empathy	Intense Empathic Experience
JOV_10	Moderate Physical Sensations	Reflective Observers	High Emotional Empathy	Self-Centered Empathy	Intense Empathic Experience
JOV_11	Intense Physical Sensations	Reflective Observers	Not Classified	Self-Centered Empathy	Intense Empathic Experience
JOV_12	Moderate Physical Sensations	Reflective Observers	High Cognitive Empathy	Self-Centered Empathy	Intense Empathic Experience
JOV_13	Mild Physical Sensations	Reflective Observers	High Cognitive Empathy	Self-Centered Empathy	Intense Empathic Experience
JOV_15	Intense Physical Sensations	Empathic Guardians	High Emotional Empathy	Self-Centered Empathy	Intense Empathic Experience
JOV_19	Intense Physical Sensations	Empathic Guardians	High Emotional Empathy	Self-Centered Empathy	Intense Empathic Experience

JOV_20	Intense Physical Sensations	Empathic Guardians	High Emotional Empathy	Self-Centered Empathy	Moderate Empathic Experience
JOV_21	Intense Physical Sensations	Empathic Guardians	High Emotional Empathy	Self-Centered Empathy	Intense Empathic Experience
JOV_22	Intense Physical Sensations	Reflective Observers	High Emotional Empathy	Self-Centered Empathy	Intense Empathic Experience
JOV_24	Intense Physical Sensations	Empathic Guardians	Low Cognitive and Emotional Empathy	Self-Centered Empathy	Intense Empathic Experience
JOV_28	Intense Physical Sensations	Empathic Guardians	High Cognitive Empathy	Self-Centered Empathy	Intense Empathic Experience
JOV_29	Intense Physical Sensations	Empathic Guardians	High Cognitive Empathy	Self-Centered Empathy	Intense Empathic Experience
JOV_34	Moderate Physical Sensations	Empathic Guardians	High Emotional Empathy	Self-Centered Empathy	Moderate Empathic Experience
JOV_35	Intense Physical Sensations	Empathic Guardians	High Emotional Empathy	Other-Centered Empathy	Intense Empathic Experience
JOV_36	Intense Physical Sensations	Reflective Observers	High Emotional Empathy	Self-Centered Empathy	Moderate Empathic Experience
JOV_38	Intense Physical Sensations	Empathic Guardians	High Emotional Empathy	Other-Centered Empathy	Moderate Empathic Experience
JOV_40	Intense Physical Sensations	Reflective Observers	High Cognitive Empathy	Self-Centered Empathy	Intense Empathic Experience
JOV_41	Mild Physical Sensations	Reflective Observers	High Cognitive Empathy	Other-Centered Empathy	Moderate Empathic Experience
JOV_42	Intense Physical Sensations	Empathic Guardians	High Emotional Empathy	Self-Centered Empathy	Intense Empathic Experience
JOV_44	Moderate Physical Sensations	Empathic Guardians	High Cognitive Empathy	Other-Centered Empathy	Moderate Empathic Experience
JOV_45	Intense Physical Sensations	Empathic Guardians	High Emotional Empathy	Self-Centered Empathy	Intense Empathic Experience

5. Reference

Aiyappa, R., An, J., Kwak, H., & Ahn, Y.-Y. (2023). *Can we trust the evaluation on ChatGPT?* (arXiv:2303.12767). arXiv. http://arxiv.org/abs/2303.12767

Chen, L., Zaharia, M., & Zou, J. (2023). *How is ChatGPT's behavior changing over time?* (arXiv:2307.09009). arXiv. https://arxiv.org/abs/2307.09009

Creswell, J. W., & Clark, V. L. P. (2017). *Designing and conducting mixed methods research.* Sage publications.

Giorgi, A., Giorgi, B., & Morley, J. (2017). The descriptive phenomenological psychological method. In *The Sage Handbook on qualitative research in psychology* (pp. 176-192.). Thousand Oaks.

Ji, Z., Yu, T., Xu, Y., Lee, N., Ishii, E., & Fung, P. (2023). Towards mitigating LLM hallucination via self reflection. *Findings of the Association for Computational Linguistics:* EMNLP 2023, 1827–1843. https://aclanthology.org/2023.findings-emnlp.123/

Morse, J. M., & Niehaus, L. (2016). *Mixed Method Design: Principles and Procedures. Routledge*. https://doi.org/10.4324/9781315424538

Petitmengin, Claire. (2006). Describing one's subjective experience in the second person: An interview method for the science of consciousness. Ph*enomenology and the Cognitive Sciences*, 5(3), 229–269.

Saunders, B., Kitzinger, J., & Kitzinger, C. (2015). Anonymising interview data: Challenges and compromise in practice. *Qualitative Research,* 15(5), 616–632.

Van Dis, E. A., Bollen, J., Zuidema, W., Van Rooij, R., & Bockting, C. L. (2023). ChatGPT: Five priorities for research. *Nature*, 614(7947), 224–226.

Van Manen, M. (2017). Phenomenology in Its Original Sense. Qualitative Health Research, 27(6), 810–825. https://doi.org/10.1177/1049732317699381

Zhang, M., Press, O., Merrill, W., Liu, A., & Smith, N. A. (2023). *How language model hallucinations can snowball* (arXiv:2305.13534). arXiv. https://arxiv.org/abs/2305.13534
